# Exploring Feature Preferences for a Treatment-Accompanying App in Patients Undergoing Radiation Therapy: Cross-Sectional Study

**DOI:** 10.2196/68411

**Published:** 2025-10-15

**Authors:** Rieka von der Warth, Nils Henrik Nicolay, Harald Binder, Melanie Boerries, Daniela Zöller, Anca-L Grosu, Erik Farin-Glattacker

**Affiliations:** 1 Section of Health Care Research and Rehabilitation Research, Institute of Medical Biometry and Statistics, Medical Center - University of Freiburg, Faculty of Medicine, University of Freiburg, Hugstetter Str. 49, Freiburg, 79106, Germany, 49 761 270 74470; 2 Institute of Medical Biometry and Statistics, Medical Center - University of Freiburg, Faculty of Medicine, University of Freiburg, Freiburg, Germany; 3 Department of Radiation Oncology, University of Leipzig, Medical Center, Leipzig, Germany; 4 Institute of Medical Bioinformatics and Systems Medicine, Medical Center - University of Freiburg, Faculty of Medicine, University of Freiburg, Freiburg, Germany

**Keywords:** patient preferences, radiation therapy, mHealth apps, app features, Digital Cancer therapy

## Abstract

**Background:**

Mobile health (mHealth) apps are playing an increasingly important role in health care, including in radiotherapy. However, adherence remains low. One way to increase adherence is to tailor app features to the patients’ preferences.

**Objective:**

This study aimed to explore the importance of patient preferences regarding the features of a therapy-supporting app in radiotherapy. In addition, we examined factors associated with the perceived importance of these features.

**Methods:**

A cross-sectional questionnaire study was conducted with patients undergoing radiotherapy between summer 2021 and winter 2022. The subjective importance of 18 features of a treatment-accompanying app was explored using a 5-point Likert scale from 1=not so important to 5=extremely important. Descriptive analyses were used to show the rated importance of app functions. Associations with possible predictors were examined using multiple hierarchical regressions, with age (interval-scaled), gender (dichotomous), previous experience with mHealth apps (dichotomous), education (3-level nominal), and supportive care needs (interval-scaled) as predictors.

**Results:**

A total of 84 radiotherapy patients participated. The average age was 62 (SD 12.5) years. The feature with the highest importance was security against hacking (46/77, 60% extremely important). Explained variances in the regression analyses ranged between *R*^2^=0.25 (The app should give me tips on suitable sporting activities that are possible with my illness) and *R*^2^=−.07 (The app should provide me with information about suitable self-help offers). Previous mHealth usage predicted the importance of 6 features, such as managing appointments (*β*=.275; *P*<.05). Decreasing age was related to 6 features, for example, showing test results and laboratory values (*β*=−.358; *P*<.05). Other predictors were an increasing age and greater supportive care needs.

**Conclusion:**

Patients undergoing radiotherapy rated app features as having varying levels of importance. The findings may help to tailor mHealth apps in radiotherapy, potentially improving adherence to app usage.

## Introduction

The use of mobile technologies is on the rise, with around 4.7 billion smartphone users worldwide [1]. It is therefore no surprise that an increasing number of mobile health (mHealth) apps are available. mHealth is defined as digital technologies offering health services [2,3]. The main uses of mHealth apps for cancer patients are, interalia, disease management, monitoring, supporting healthy behavior, and interpersonal exchange with other patients [2,4]. mHealth apps have shown a high degree of feasibility and patient acceptance among patients with cancer [5] including early detection of cancer relapse [6]. At the same time, more and more health care providers are offering mHealth solutions to accompany treatments and health services, such as providing information on floor plans of provider sites, individual appointment planning, and legal, medical documents. As an example, the Medical Center – University of Freiburg provides the “Meine Uniklinik” app [My university medical center] for patients and visitors, helps them navigate the hospital campus and their treatment process [7]. Overall, these apps aim to enhance treatment processes and increase patient satisfaction, and thus potentially improve treatment outcomes in the long run [6].

However, many mHealth apps show insufficient patient usage, as patients often stop using the app shortly after download, do not follow usage recommendations, or never use it at all—even when prescribed by doctors [8,9]. This nonusage has been shown to be associated with the limited effects of mHealth interventions [8,10]. Thus, tailoring mHealth apps to individual patient needs has recently gained importance in order to increase usage and app adherence [11,12]. A recent review has found no clear evidence of consistent predictors of mHealth use in patients with cancer [12], even though some research suggests that female gender, increasing age, and a higher education are related to greater usage [13-16]. Other predictors included the number of comorbidities or the experience of pain [12]. Given the unclear evidence on usage predictors, the question of what factors might positively influence usage remains open.

One promising approach to tailoring mHealth apps might be to consider patient preferences for mHealth app features. One study found that the flexibility of mHealth apps to adapt to patient preferences positively influences patients’ expectations of app performance [17]. Considering patient preferences is also known to be a key aspect in providing patient-centered care [18,19]. Since care coordination is essential to patient-centeredness [19], which could also be provided through treatment-accompanying apps, understanding patient preferences is relevant. Furthermore, by addressing patient preferences related to care processes, treatment outcomes might be positively affected [18]. However, little is known about patient preferences for mHealth apps accompanying treatment in patients with cancer [20], even though detecting patients’ needs before developing a mHealth app is relevant to developing targeted measures [21].

In conclusion, the aim of this study was to explore patient preferences for app features and to investigate the possible associated factors. Specifically, we aimed to explore the following research questions: (1) How do patients undergoing radiation therapy rate the importance of the features of an app accompanying treatment? (2) What factors are associated with the importance ratings of the features?

We conducted a quantitative cross-section study. The questionnaire used was based on findings from a qualitative preliminary study. To examine associations, the following factors were investigated: gender, previous use of mHealth apps, education level, age, and supportive care needs.

## Methods

### Ethical Considerations

This study was conducted at the Medical Center - University of Freiburg. Ethical approval was granted by the University of Freiburg Ethics Council (approval number: 377/19), and the study was registered in the German Clinical Trial Register (DRKS00020362). Written informed consent was obtained from all participants in the preliminary and main studies. Participants received no compensation for their participation. All data were pseudonymized to ensure confidentiality.

### Preliminary Study and Questionnaire Development

We conducted a qualitative preliminary study in the summer of 2020 with a total of 4 patients. The number of participants was determined based on practical considerations during the COVID-19 pandemic, where contact was to be limited. Included patients were those visiting the Department of Radiation Oncology at the Medical Center – University of Freiburg for their initial consultation before the start of radiation therapy. The aim of the preliminary study was to assess patients’ feature preferences for a mobile app accompanying hospital treatments. For this purpose, qualitative interviews were conducted using a semistructured interview guideline. The guideline began with an open-narrative question, asking the participant to elaborate on their expectations of a treatment app before the initial consultation, immediately after the initial consultation, during treatment, and after the completion of treatment. Afterward, patients were shown an early version of the “Meine Uniklinik” app, a treatment accompanying app developed by the Medical Center – University of Freiburg [7]. Patients were given some time to use the app before the interview proceeded. They were then asked about their first impression of the app and its features, and whether the app aligns with their previously matched expectations. All the interviews were digitally recorded and transcribed verbatim by an external service provider.

**Figure 1.**
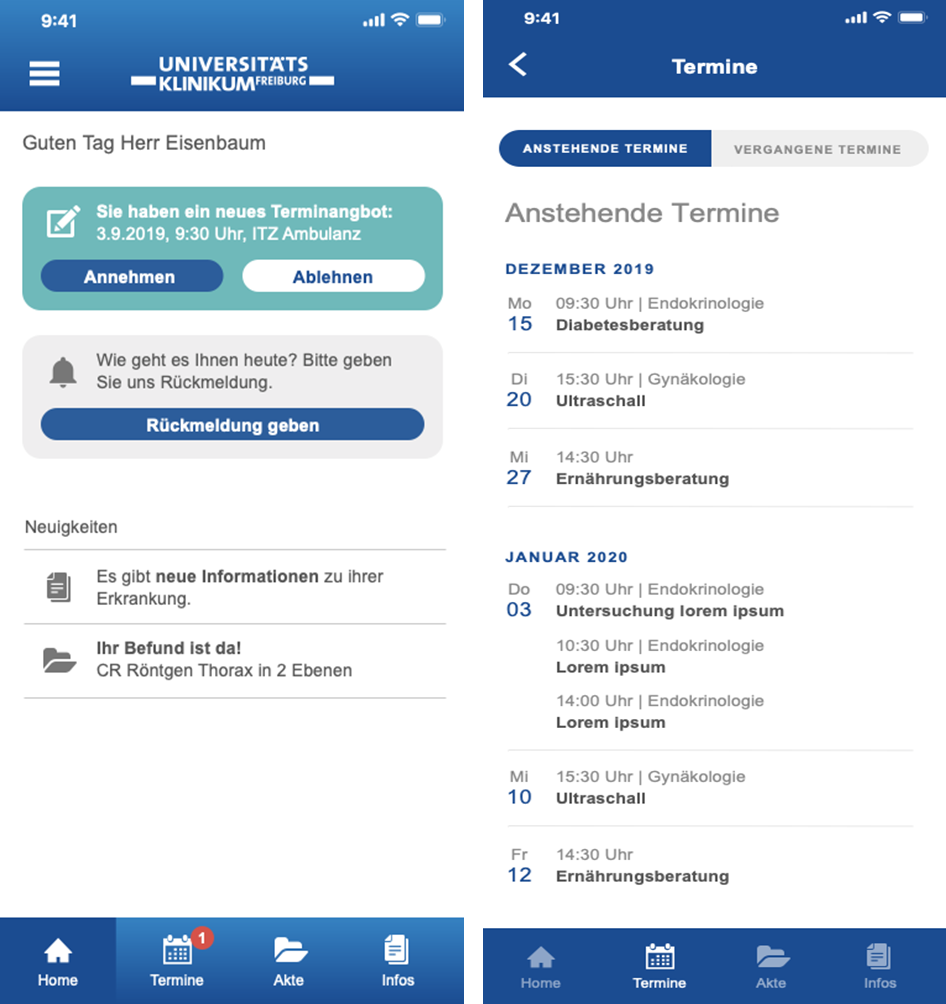
Screenshots of the “Meine Uniklinik” app used during the study showing the home screen and the overview of appointments.

We categorized the interview content into different categories. The categories comprised the treatment organization, the therapy process, therapy participation, information about the clinic and providers, emotional support, services, design, and concerns. Based on these categories, items in respect to patient preferences for a mobile app accompanying hospital treatment were developed. For this purpose, one of the authors (RW) developed an initial version of items, aiming to create one item for each feature mentioned by participants. Two of the other authors (EFG and NHN) reviewed the items, with a specific focus on comprehensibility and unidimensionality. Finally, a total of k=18 items were developed as regards feature preferences related to, interalia, the organization of treatment, appointments, and networking with other patients. Example items include “The app should give me information about the therapy process” and “The app should remind me of my follow-up appointments.” Answers were given on a 5-point Likert scale from 1=not so important to 5=extremely important. The questionnaire began with pictures of the “Meine Uniklinik” app to give participants an impression of what such an app might look like (refer to Figure 1). For the purpose of publication, the questionnaire items were translated into English by the author of this manuscript.

The questionnaire was tested with a total of 5 participants. Attending doctors asked these participants for feedback, in particular as to whether all the items were understandable and if the length of the questionnaire was acceptable. All 5 participants rated the questionnaire as acceptable in both length and clarity. Since all the participants rated the questionnaire as acceptable and no changes were made, the 5 participants were included in the final sample.

### Further Assessments: Supportive Care Needs

To assess the supportive care needs of participants, the Short-Form Supportive Care Needs Survey [21] in its 9-item short form as described by Girgis and Stojanovski [22], was used. We used supportive care needs as possible associative factors, hypothesizing that the app might be able to provide supportive care. The 9 items describe the psychological, health systems and information, daily living, patient care, and sexuality domains as in the original form. The brief 9-item version is efficient and reliable for clinical samples that have limited time [23]. For analysis, a mean score is calculated and converted to a 0‐ to 100-point scale, with 100 indicating greater supportive care needs. Cronbach α was 0.87 in this study.

### Sociodemographic Data

Sociodemographic data included gender, age, highest school education, employment status, and previous usage of mHealth apps.

### Recruitment of Participants

As part of the study, we aimed to recruit 200 participants during their initial consultations at the Department of Radiation Oncology, Medical Center – University of Freiburg, between summer 2021 and winter 2022. A sample size of 200 was deemed sufficient to explore associations between various factors and the perceived importance of app features. In total, we successfully recruited 84 participants, corresponding to a recruitment rate of 42%. Eligible patients had to be of legal age (≥18 y) and have sufficient German language skills. Attending doctors led the recruitment process by informing potential patients about the study and handing out study information and the questionnaire. Both the study information and questionnaire were paper-based. Participants were allowed to complete the questionnaire directly after the consultation while still at the clinic, or at home, and to then return it at the following appointment. Participants took about 20 minutes to complete the questionnaire. Final questionnaire data was entered into the database using an automated scanning process with a PDF to Microsoft Excel step.

We have no information on patients who were eligible and declined to participate.

### Analysis: Description of Feature Preferences

Analyses were conducted at the level of the individual item. First, we conducted a descriptive analysis of each item, describing the subjective importance of the corresponding feature.

### Associations With Feature Preferences

Associations with possible predictors were examined using multiple hierarchical regression analyses, with the following factors as predictors: Gender (dichotomous: female or male), experience with mHealth apps (dichotomous: yes or no), highest level of education (dummy-coded), age (continuous variable), and supportive care needs (continuous variable) were chosen as possible associated factors. Assumptions for all the regression models were tested by checking the value of the variance inflation factor, the Durbin-Watson statistic, plots of *ZRESID against *ZPRED and normal P-P plots. All the assumptions were met. All the analyses were conducted using IBM’s SPSS Version 29 [22].

## Results

### Participants

Although 84 patients submitted their questionnaires, data were available in only 82 cases, as 2 participants returned empty questionnaires. One person identified as gender diverse and was therefore excluded from further analyses in view of the small number, resulting in a final sample of 81 patients. Most participants were male (41/78, 52% responses), the mean age was 62 (SD 12.5) years. About 31% (25/81) of the participants had previously used a mHealth app. More information on the participants can be found in Table 1.

**Table 1.** Sociodemographic data of participants (N=81).

		Values	
Gender, n (%)			
	Male	41 (53)	
	Female	37 (47)	
Living with a partner, n (%)			
	Yes	56 (72)	
	No	22 (28)	
Employment status, n (%)			
	Yes	35 (44)	
	Housewife or man	2 (3)	
	Unemployed	1 (1)	
	Disability pension	5 (6)	
	Pension	36 (45)	
	Others	1 (1)	
Highest school education, n (%)			
	Basic secondary school (German: Volksschul-oder Hauptschulabschluss)	30 (38)	
	Secondary school (German: Mittlere Reife or Realschule)	24 (30)	
	High school (German: Abitur or Fachhochschulreife)	25 (31)	
	Other	1 (1)	
Previous usage of an mHealth^a^ app, n (%)			
	Yes	25 (31)	
	No	56 (69)	
Age (years), mean (SD); range		62.0 (12.5); 28.0‐84.0	
Supportive care needs^b^, mean (SD); years		44.8 (23.3); 0.0‐92.6	

a mHealth: mobile health.

b Score between 0-100, higher score indicating higher needs.

### Description of Feature Preferences

Participants showed a high diversity in their preferences for app features. For each item, responses were received from up to 77 out of 81 study participants. Overall, 46 out of 77 (60%) participants rated safety against hacker attacks as extremely important, whilst sending notifications about changes in treatment was rated as extremely important by 33 out of 77 (43%) participants. The feature rated least important was the option to network with other patients, with 39 out of 76 (51%) participants rating it as not important. An overview of all items can be found in Table 2.

**Table 2.** Importance ratings of features preferences (N=81).

The app should…	Patients, n	Not so important (1), n (%)	Somewhat important (2), n (%)	Important (3), n (%)	Very important (4), n (%)	Extremely important (5), n (%)	Missings, n (%)
… be absolutely safe from attacks by “hackers.”	77	4 (4.9)	2 (2.5)	13 (16.0)	12 (14.8)	46 (56.8)	4 (4.9)
… send me messages if something changes for me.	77	4 (4.9)	—^a^	16 (19.8)	24 (29.6)	33 (40.7)	4 (4.9)
… should remind me of my follow-up appointments	76	5 (6.2)	3 (3.7)	21 (25.9)	23 (28.4)	24 (29.6)	5 (6.2)
… show me my test results and laboratory values.	76	4 (4.9)	3 (3.7)	34 (42.0)	17 (21.0)	18 (22.2)	5 (6.2)
… offer me the opportunity to manage my appointments.	77	9 (11.1)	10 (12.3)	17 (21.0)	23 (28.4)	18 (22.2)	4 (4.9)
… be intuitive to use.	75	5 (6.2)	4 (4.9)	24 (29.6)	25 (30.9)	17 (21.0)	6 (7.4)
… give me information about the timing of the therapy.	76	5 (6.2)	4 (4.9)	23 (28.4)	28 (34.6)	16 (19.8)	5 (6.2)
… be designed attractively.	75	7 (8.6)	5 (6.2)	37 (45.7)	13 (16.0)	13 (16.0)	6 (7.4)
… give me information about the therapy process.	75	5 (6.2)	4 (4.9)	26 (32.1)	28 (34.6)	12 (14.8)	6 (7.4)
… have an integrated taxi call so that I can arrive and depart easily.	76	25 (30.9)	16 (19.8)	13 (16.0)	12 (14.8)	10 (12.3)	5 (6.2)
… address me personally, eg,via an individual start image.	76	24 (29.6)	20 (24.7)	16 (19.8)	9 (11.1)	7 (8.6)	5 (6.2)
… give me tips on suitable sporting activities that are possible with my illness.	76	14 (17.3)	12 (14.8)	29 (35.8)	15 (18.5)	6 (7.4)	5 (6.2)
… give me an overview of the location of the rooms in the clinic.	76	8 (9.9)	20 (24.7)	27 (33.3)	15 (18.5)	6 (7.4)	5 (6.2)
… offer me help in dealing with therapy-related anxiety.	76	18 (22.2)	15 (18.5)	27 (33.3)	12 (14.8)	4 (4.9)	5 (6.2)
… motivate me to actively participate in the therapy.	75	12 (14.8)	17 (21.0)	33 (40.7)	10 (12.3)	3 (3.7)	6 (7.4)
… inform me about a healthy diet.	74	15 (18.5)	18 (23.5)	25 (32.1)	15 (18.5)	1 (1.2)	5 (6.2)
… give me the opportunity to network with other patients.	76	39 (48.1)	24 (29.6)	6 (7.4)	7 (8.6)	—	5 (6.2)
… provide me with information about suitable self-help offers.	76	18 (22.2)	27 (33.3)	26 (32.1)	5 (6.2)	—	5 (6.2)

a Not applicable.

### Associations With Feature Preferences

Explained variances in the analyses ranged between *R*^2^=0.25 (The app should give me tips on suitable sporting activities that are possible with my illness) and *R*^2^=−.07 (The app should provide me with information about suitable self-help offers), indicating a limited fit of the predictors in some models. Gender and highest education were not significantly related to any preferences.

Previous mHealth app usage was associated with higher importance for 6 preferences. Participants who had previously used mHealth apps stated placing greater importance on the app offering the opportunity to manage appointments (*β*=.275; *P*<.05), and having a message feature if something changes (*β*=.342; *P*<.05) as well as an integrated tax call (*β*=.315; *P*<.05). Furthermore, participants with previous mHealth app usage placed greater value on functions concerning the showing of test results and laboratory values (*β*=.398; *P*<.01), the offering of information about a healthy diet (*β*=.575; *P*<.001), and the offering of suitable tips for sporting activities (*β*=.500; *P*<.001).

Age was related to higher importance in eight preferences in total. With decreasing age, participants had a greater need for the opportunity to manage appointments (*β*=−.459; *P*<.01), having information about therapy processes in the app (*β*=-.401; *P*<.05) as well as a feature showing test results and laboratory values (*β*=−.358; *P*<.05). Moreover, the app should further send messages if something changes (*β*=−.491; *P*<.01). In addition, it was more important to them that the app was attractively designed (*β*=−.372; *P*<.05) and should be intuitive to use (*β*=−.446; *P*<.01).

In contrast, increasing age was associated with the greater importance of 2 features: the app giving information about the timing of the therapy (*β*=.522; *P*<.001) and also reminding participants about follow-up appointments (*β*=.597; *P*<.001).

Finally, having higher supportive Care Needs was associated with a greater need for the app to address the patient personally (*β*=.367; *P*<.01). An overview of all the analyses is given in Table 3.

**Table 3.** Associations with preferences of app features.

The app should...	Patients, n	Gender^a^, standard β (*P* value)	Previous usage of an mHealth app^b^, standard β (*P* value)	Highest education (intercept=basic secondary school)		Age, standard β (*P* value)	Supportive care needs, standard β (*P* value)	*R*^2^/ corrected *R*^2^
				Secondary school, standard β (*P* value)	High school, standard β (*P* value)			
...offer me the opportunity to manage my appointments.	56	.086 (.655)	.275 (.046)^c^	−.172 (.320)	−.265 (.129)	−.459 (.003)^c^	.081 (.582)	0.31/0.23
...give me information about the timing of the therapy.	56	−.086 (.657)	.236 (.085)	.022 (.900)	−.118 (.494)	−.522 (<.001)^c^	−.247 (.097)	0.32/0.23
...give me information about the therapy process.	56	−.211 (.323)	.160 (.286)	.137 (.473)	−.115 (.546)	−.401 (.018)^c^	.248 (.131)	0.16/0.06
...show me my test results and laboratory values.	56	−.185 (.36)	.398 (.007)^c^	−.003 (.99)	−.273 (.14)	−.358 (.03)^c^	.226 (.15)	0.24/0.15
...inform me about a healthy diet.	56	–.121 (.54)	.575 (<.001)^c^	−.028 (.87)	−.225 (.20)	−.071 (.64)	.031 (.84)	0.30/0.21
...give me tips on suitable sporting activities that are possible with my illness.	56	.025 (.89)	.500 (<.001)^c^	.065 (.70)	−.069 (.68)	−.190 (.20)	−.088 (.54)	0.35/0.27
...give me an overview of the location of the rooms in the clinic.	56	−.144 (.51)	.082 (.60)	.121 (.54)	−.095 (.63)	−.312 (.07)	.197 (.25)	0.10/–0.01
...motivate me to actively participate in the therapy.	55	.218 (.31)	.184 (.22)	−.002 (.99)	−.203 (.30)	−.162 (.34)	−.103 (.53)	0.16/0.06
...offer me help in dealing with therapy-related anxiety.	56	.069 (.75)	.242 (.11)	−.068 (.72)	−.358 (.07)	−.217 (.20)	.040 (.81)	0.16/0.05
...should remind me of my follow-up appointments.	56	−.103 (.59)	.137 (.32)	−.076 (.66)	−.167 (.34)	−.597 (<.001)^c^	.181 (.23)	0.31/0.22
...have an integrated taxi call so that I can arrive and depart easily.	56	−.207 (.34)	.315 (.04)	−.100 (.60)	−.074 (.70)	−.212 (.21)	.311 (.06)	0.16/0.05
...give me the opportunity to network with other patients.	56	.013 (.95)	.046 (.77)	-.018 (.93)	−.013 (.95)	−.078 (.66)	−.206 (.24)	0.05/−0.06
...provide me with information about suitable self-help offers.	56	−.075 (.74)	.150 (.35)	.010 (.96)	−.022 (.92)	−.190 (.28)	.018 (.92)	0.05/–0.07
...be designed attractively.	56	−.110 (.61)	.069 (.65)	.132 (.50)	.049 (.80)	−.372 (.03)^c^	.048 (.77)	0.14/0.03
...be intuitive to use.	55	−.044 (.83)	−.028 (.84)	.174 (.34)	.230 (.20)	−.446 (.006)^c^	−.037 (.81)	0.29/0.21
...send me messages if something changes for me.	56	−.296 (.15)	.342 (.02)^c^	.014 (.94)	−.051 (.78)	−.491 (.003)^c^	.230 (.14)	0.25/0.16
...address me personally, eg, via an individual start image.	55	−.324 (.16)	.138 (.38)	.282 (.16)	.054 (.78)	−.129 (.46)	.367 (.04)^c^	0.11/0.00
...be absolutely safe from attacks by “hackers.”	56	−.216 (.33)	.243 (.12)	.244 (.22)	.120 (.55)	−.178 (.30)	.111 (.51)	0.09/−0.02

a Gender: 1=male/2=female.

b Previous app usage: 0=no and 1=yes.

c Significant.

## Discussion

### Principal Findings

In this study, we explored patient preferences in respect of the features of a mHealth app accompanying treatment in a university hospital in Germany. Overall, participants in our study rated the features with varying degrees of importance, with the most crucial feature being security against hacker attacks. Other highly rated features related to the process and organization of the treatment, such as the administration and timing of appointments. Decreasing and increasing age, and previous usage of mHealth apps seem to be associated with greater importance being attributed to specific features. Understanding patient preferences might be relevant for increasing the usage of such apps, potentially leading to improvements in treatment processes and health outcomes. Furthermore, as patient opinions are often not considered during the development of mHealth apps in cancer care [20], the results we presented might help to encourage further apps’ development. Recent literature shows that tailoring mHealth apps to patient preferences does increase engagement rates in cancer care [24].

### Comparison to Previous Work

Overall, participants rated features as being of differing importance, with the most important feature being security from hacker attacks. Other highly rated features related to information on the process and organization of the treatment, such as the administration and timing of appointments. This is hardly surprising, as we did include patients at their initial consultation and there is some evidence indicating that cancer patients have a great need for information about therapy contents and processes at the beginning of therapy [25]. In contrast, participants noted a limited need to connect with other patients or to receive information on self-help resources. This is remarkable as self-help offers and peer-support groups are generally perceived as helpful [26] and are often included in mHealth apps [27]. Interestingly, patients in a previous study concerning the “Meine Uniklinik” app requested this specific feature for a mHealth app in oncology [28]. It is possible that participants might perceive a treatment-accompanying app to not be an appropriate platform for promoting these services. However, due to the exploratory nature of our study, we cannot draw a conclusion about self-help resources in mHealth apps.

In line with the literature, we found that both increasing and decreasing age groups could be related to the feature importance, whilst there are contrasting results with regard to age [12,29]. In fact, the relationship between age and the adoption of mHealth apps seems to be complicated, with the perceived ease of use becoming more important with age [30]. Surprisingly, we ascertained that a decreasing, rather than an increasing age, was associated with a need for intuitive use and attractive design. Given that both preferences associated with an increasing age are related to the timing and therapy schedule, one might assume that this is the most important need for older patients. Furthermore, we found in our sample that experiences with health apps are related to a greater importance of different kinds of features, for example, the organization of the treatment and information on the illness itself (test results or diet tips). One could hypothesize that, due to previous experiences, participants place greater importance on those features when they have already encountered and therefore know which features are beneficial, or where they are able to transfer previous knowledge of the features to another app.

Notably, higher supportive care needs were only related to the greater importance of the app being personalized, even though the questionnaire assesses concepts like the need for information on laboratory values or behavior changes to increase the quality of life [23,31], which we also included as features in our study. One possible reason might be that patients do not expect these needs to be met by a treatment-accompanying app. Alternatively, the limited sample size may have contributed to supportive care needs being significantly related to only one feature.

As Woldeyohannes and Ngwenyama [17] stated, the flexibility of features is a key factor in influencing app usage. This is in line with the theory on patient preferences, which states that patient preferences are not a stable concept and do change over time [18]. Thus, even though we assessed preferences at only one measurement point, we assume that the preferences for features in a treatment-accompanying app will change during the course of treatment. It is therefore important for the usage and uptake of a treatment-accompanying app for it to be flexible with the features and for these to be adjustable by the patients.

### Strengths and Limitations

In this cross-sectional study, we explored patients’ preferences for a treatment-accompanying app in radiation therapy. Knowledge about patients’ preferences is of relevance to improve usage of mHealth apps and to thus enhance treatment processes. The results might be used to design treatment-accompanying apps for patients undergoing radiation therapy and could thus possibly influence clinical decision-making.

First, we should discuss the assessment of the app features. We based our assessment of features on a qualitative preliminary study to assess only those features relevant to patients. Both patient groups in the preliminary study and main study were briefed either by screenshots of an example app, or the use of an early version of the “MeineUniklinik” app. This could have led to positive and negative effects on the assessment, either by limiting answers to the displayed features or helping patients imagine a corresponding app. For instance, patients had previously stated that a symptom and side effect tracker would be of use [20]. However, no patients in our preliminary study considered this feature to be necessary. Thus, the features assessed in this study require further validation.

Second, we were not able to gather any medical information on our participants, and this has proved to be an important disadvantage of our study. This was due to the COVID-19 pandemic, where no medical resources were available to assess patients’ information. However, medical information should be considered in the future, as previous research suggests it is associated with app usage patterns [12].

Finally, the most important limitations are the small sample size, as well as the cross-sectional nature of the study. The latter is especially relevant considering the time instability of patient preferences. Furthermore, due to the small sample size, we decided not to control for multiple testing. However, this limits the interpretation of our results. Future research should focus on the assessment of feature preferences in therapy-accompanying apps using a longitudinal design, as well as focus on bigger sample sizes. These studies should also investigate the time instability of preferences, where possible.

### Conclusion

In this paper, we explored how patients undergoing radiotherapy assess the importance of mHealth app features. The most important feature was security from hacker attacks, according to our sample. Age and previous usage of mHealth apps seem to be associated with the greater importance of different features. The findings in this study might be used to develop future apps after further validation, allowing tailoring to the individual patient and, thereby, enhancing patient-centered mobile care in radiation oncology. In this way, treatment processes might be improved, which in turn, might impact treatment outcomes.

## Abbreviations

mHealth: mobile health
